# Use of the psychomotor vigilance test to aid in the selection of risk controls in an air medical transport operation

**DOI:** 10.1093/sleepadvances/zpad003

**Published:** 2023-01-17

**Authors:** Daniel J Mollicone, Kevin Kan, Sara Coats, Christopher Mott, Matthew van Wollen, Arvida Hatch, Joseph Gallagher, Sheryl Williams, David Motzkin

**Affiliations:** Pulsar Informatics, Inc., Seattle, WA, USA; Pulsar Informatics, Inc., Seattle, WA, USA; Pulsar Informatics, Inc., Seattle, WA, USA; Pulsar Informatics, Inc., Seattle, WA, USA; Pulsar Informatics, Inc., Seattle, WA, USA; PHI Health, LLC, Phoenix, AZ, USA; PHI Health, LLC, Phoenix, AZ, USA; PHI Health, LLC, Phoenix, AZ, USA; PHI Health, LLC, Phoenix, AZ, USA

**Keywords:** neurobehavioral performance, shift work, sleepiness, sleep/wake cognition

## Abstract

**Study Objectives:**

This study evaluated the utility and ecological validity of the 3-minute psychomotor vigilance test (PVT) completed by safety-critical personnel in an air medical transport operation as part of a fatigue risk management program.

**Methods:**

Crewmembers in an air medical transport operation self-administered an alertness assessment incorporating a 3-minute PVT at different time points during their duty schedule. The prevalence of alertness deficits was evaluated based on a failure threshold of 12 errors considering both lapses and false starts. To evaluate the ecological validity of the PVT, the relative frequency of failed assessments was evaluated relative to crewmember position, timing of the assessment within the duty schedule, time of day, and sleep quantity in the last 24 h.

**Results:**

2.1% of assessments were associated with a failing PVT score. Crewmember position, timing of assessment within the duty shift, time of day, and sleep quantity in the last 24 h were found to affect the relative frequency of failed assessments. Obtaining less than 7–9 h of sleep was associated with systematic increases in the failure rate (*F*[1, 54 612] = 168.1, *p* < .001). Obtaining less than 4 h of sleep was associated with a frequency of a failed assessment 2.99 times higher than the frequency of a failed assessment when obtaining 7–9 h of sleep.

**Conclusions:**

Results provide evidence for the utility and ecological validity of the PVT as well as the suitability of the PVT failure threshold to support fatigue risk management in safety-critical operations.

**Statement of Significance**

This study provides evidence for the ecological validity and utility of the psychomotor vigilance test as an objective quantitative measure of alertness to guide the application of risk controls in safety-critical operations. It is a demonstration of the real-world impact that extends from Dr David F. Dinges’s legacy of scientific discovery.

## Introduction

Dr Dinges’s development of the psychomotor vigilance test (PVT) as an objective quantitative assay of vigilant attention has provided a cornerstone tool for the sleep research community. Today, the PVT is increasingly playing a role in operational safety management [1, 2].

Pulsar Informatics, Inc. is an applied science organization that provides software to monitor for and mitigate operational fatigue risk. Pulsar has had the honor of collaborating with Dr Dinges to adapt and deliver the PVT on ubiquitous computing platforms (e.g. laptops, tablets, and smartphones) [3]. In this article, we present data from an air medical transportation operation that uses the PVT as a standard operational measure to identify situations that may require safety interventions. We highlight the fact that Dr Dinges’s contributions go far beyond the research domain to impact safety outcomes in operational settings.

In any safety-sensitive environment, workers must be reliably alert to ensure safe operations [1]. Operational factors such as restricted sleep opportunities, long duties that extend the waking period, and working at night can contribute to alertness deficits and elevated fatigue-related risks [4–6].

The PVT provides an objective, quantitative assessment of alertness deficits to aid in the detection of operational scenarios where a worker may require a risk control to be implemented to ensure safety [1]. The PVT is a computer-based test that requires users to respond to randomly timed visual stimuli [7]. Alertness deficits are quantified by sampling errors of omission (lapses) and errors of commission (false starts), as well as response slowing [2, 8]. The PVT has the minimal aptitude or learning effects, which makes it ideally suited for repeated administration in an operational environment [9, 10].

The original PVT developed by Dr Dinges is 10 min in duration; however, several variants have been developed [8, 11]. In this application, the briefer 3-minute version is used as it presents a smaller time burden on mission-critical operational personnel. The 3-minute PVT has modestly reduced sensitivity compared to the 10-minute version [11].

The PVT was incorporated as a standard operational measure to evaluate the alertness of mission-critical personnel at PHI Health, LLC, a provider of air medical transportation services. In this organization, mission-critical crewmembers include pilots, flight paramedics, flight nurses, mechanics, and Operational Control Center (OCC) personnel. An air medical transport response team, typically comprised of a pilot, a flight paramedic, and a flight nurse, is assigned to a base of operation. Crewmembers work a range of different duty schedules based on role. In accordance with FAA regulations, pilots are typically assigned the following duty pattern: seven consecutive 12-hour night duties, followed by 1 week off duty, followed by 7 consecutive 12-hour daytime duties, followed by 1 week off duty. Flight paramedics and flight nurses typically work 24-hour duty periods, normally followed by one or more days off duty. Mechanics typically work seven consecutive duty periods that are 8–12 h in duration, followed by 7 days off duty. Mechanics are additionally required to provide on-call coverage outside normal duty hours. OCC personnel typically work four 12-hour duty shifts, followed by 3 days off duty.

Each air base is equipped with sleeping facilities, and the naturally occurring pattern of air transport calls normally provides crewmembers with adequate rest opportunities while on duty. If at any time a crewmember perceives that they are not fit for duty for any reason, they may exercise well-established protocols to remove themselves from duty without personal jeopardy until the fitness for duty concern is resolved.

Introducing the PVT to an operation to augment safety assurance procedures typically follows a two-step implementation process: a benchmarking phase and an active intervention phase. The benchmarking phase typically lasts 6–18 months. Its purpose is to evaluate the ecological validity of failure thresholds and to generate estimates of the frequency that risk controls will be required in the active intervention phase. During the benchmarking phase, crewmembers complete alertness assessments and receive immediate feedback about their results. If based on a specific assessment result, a crewmember perceives that they are not fit for duty, they are instructed to follow an established protocol to temporarily remove themselves from duty. No other organizational risk controls are used based on PVT assessment results during the benchmarking phase.

Based on an analysis of data collected during the benchmarking phase, the organization identifies a set of risk controls to be implemented in the case of adverse assessment results. Risk controls may include substituting the crewmember or taking the base out of service for one or more hours to enable the crewmember to obtain rest and restore alertness levels. In this article, we present alertness assessment data from the benchmarking phase.

## Methods

**Figure 1.**
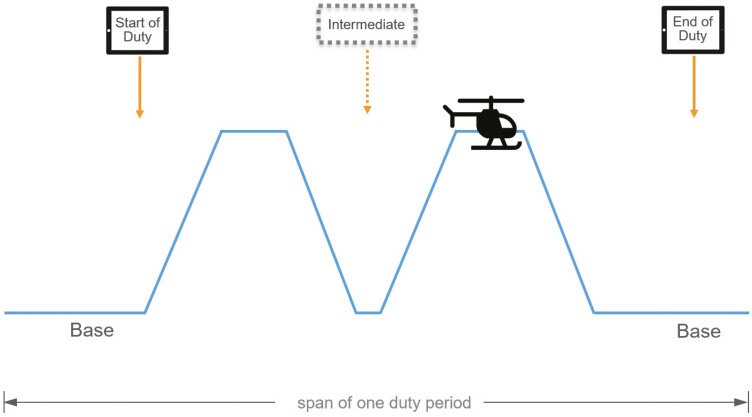
Schematic illustration showing the timing of assessments relative to the duty period. Crewmembers were instructed to self-administer an alertness assessment incorporating a 3-minute PVT (PVT-B) within 30 min of commencing a duty period, within 30 min of completing the duty period, and at intermediate time points during the duty period following transports that occurred at night (between 12:00 am and 6:00 am). The timing of the testing was the same, irrespective of shift type, for each crewmember position.

Crewmembers were instructed to self-administer an alertness assessment incorporating a 3-minute PVT (PVT-B) within 30 min of commencing a duty period, within 30 min of completing the duty period, and at intermediate time points during the duty period following transports that occurred at night (between 12:00 am and 6:00 am). See Figure 1.

The PVT was conducted using PVT WorkFit, a native software application for Apple iPad developed by Pulsar Informatics. In this version of the PVT, the participant simply taps the screen of the iPad when the numerical visual stimulus appears in a rectangle in the center of the screen. Crewmembers were instructed to complete assessments during their duty period when they did not have to perform operational duties associated with transports. Crewmembers were instructed to find a quiet location around the base in a location free from distraction (e.g. away from others that may be talking or from a television set).

PVT results data were uploaded to servers maintained by Pulsar Informatics and then deidentified for the purpose of analysis. Results were expressed in terms of the number of lapses (responses longer than 355 ms), false starts (premature responses), and the total number of errors (lapses plus false starts).

A failure threshold of 12 total errors on an assessment was selected, consistent with other implementations of the PVT in occupational settings and based on sleep deprivation studies in the literature that documented PVT performance as a function of partial and total sleep deprivation [2, 4, 11]. The rationale was to select a failure threshold below which alertness deficits would normally not be observed for fit adults in typical working conditions. Scores of 8 to 11 total errors are approximately the range observed during night duty, after being awake more than 16 h, after the fifth day of daily restricted sleep opportunities restricted to 4 h, or when having a blood alcohol concentration of 0.04 g/dL [4–6].

Crewmembers were provided training to aid in the interpretation of assessment results. After viewing assessment results, if a crewmember determined that they were not fit for duty, they were instructed to follow established protocols to temporarily remove themselves from duty. No other organizational risk controls based on PVT assessment results were utilized during the benchmarking phase of implementation.

All assessments performed by crewmembers were time-stamped. In addition to the PVT-B, each assessment also collected information related to crewmember position (pilot, flight paramedic, flight nurse, mechanic, or OCC specialist) and the timing relative to duty period (within 30 min of the start of duty, intermediate timepoint during duty, within 30 min of the end of duty). For assessments delivered at the start of duty, crewmembers were also asked how much sleep (in hours and minutes) they had obtained in the last 24 h.

A total of 1061 crewmembers provided 100 674 alertness assessments during the period of January 18, 2019 through August 25, 2020. On average each crewmember provided 94.6 assessments (range: 1–560, *SD* = 107.2). Assessments that had too many false starts (>30 false starts or >12 consecutive false starts), all timeouts, or average RT > 1 s were flagged as having data quality issues. 3.1% of all assessments were determined to exhibit data quality issues and were excluded from the analysis.

Further, the first two assessments completed by each crewmember (familiarization assessments) were determined to significantly impact the failure rate (10.6% compared with 2.1% overall; *t* (1966) = 20.88, *p* < .001); these assessments were also excluded from analysis, accounting for an additional 2.1% of the total data. Thus, 5.2% of the data were excluded from analysis.

**Figure 2.**
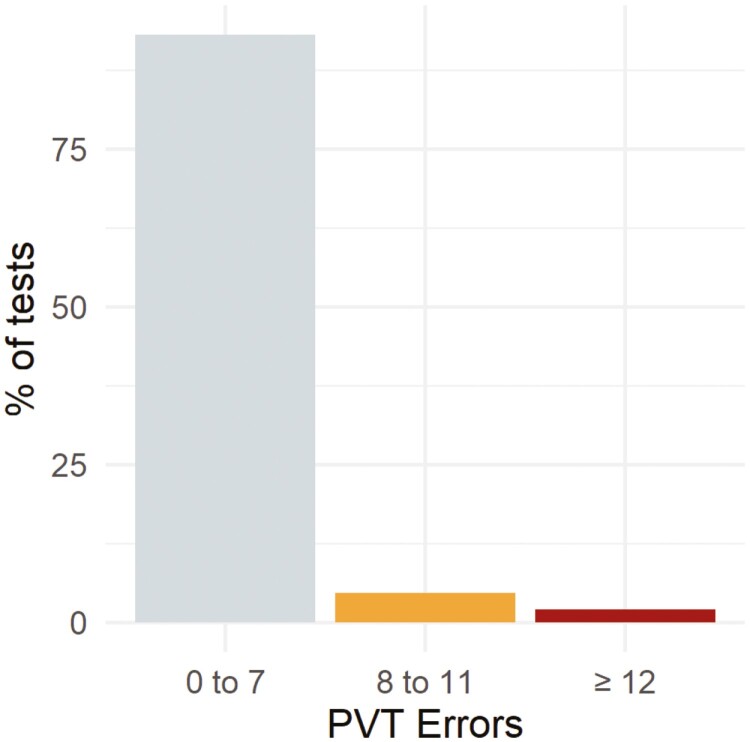
Histogram of 3-minute PVT assessments completed during the benchmarking phase of a fatigue risk management program. These data help quantify the prevalence of failed assessments as a preparatory step for the active intervention phase of the fatigue risk management program, during which a risk control will be applied in response to a failed assessment. 93.2% of assessments resulted in a nominal score (0–7 total errors), 4.6% of assessments resulted in an intermediate score indicating moderate alertness deficits (8–11 total errors), and 2.1% of assessments resulted in a failed score (12 or more total errors).

A total of 95 493 assessments meeting quality control criteria were evaluated. 56 947 assessments were associated with the start of a duty period, 6938 assessments were completed during a duty period, and 31 608 assessments were associated with the end of a duty period. Figure 2 shows a histogram of the assessments by the total number of errors (lapses plus false starts).

To evaluate the ecological validity of assessments, several factors that would be expected to affect the failure rate were considered, including (1) crewmember position, (2) assessments occurring during nighttime hours (12:00 am to 6:00 am), (3) timing of the assessment within the duty period, and (4) self-reported sleep quantity obtained in the last 24 h. Notably, other confounding factors known to impact PVT assessments, including age and sex [12], were not available in the data and therefore not considered in the analysis.

## Results

**Figure 3.**
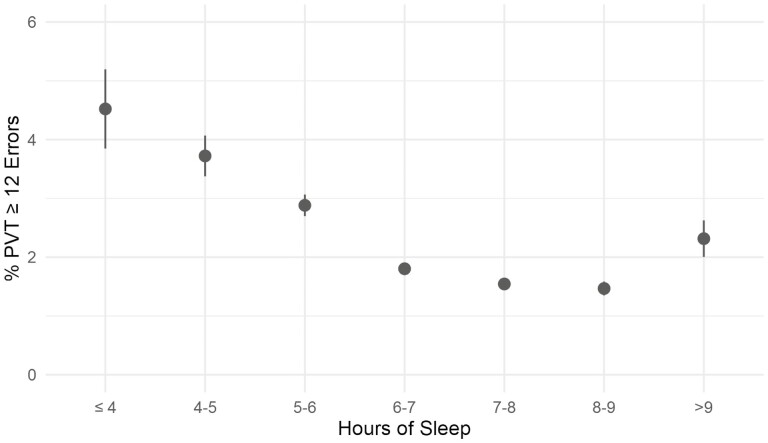
Relative frequency of failed 3-minute PVT assessments completed by mission-critical crewmembers in an air medical transport operation expressed as a function of self-reported sleep quantity obtained in the last 24 h. The failure threshold was set at 12 total errors, being the sum of lapses (>355 ms) and false starts. Obtaining less than 7–9 h of sleep was associated with increased failure rates. Each hour of sleep loss below 7–9 h of sleep accounted for an average 0.7% increase in the relative frequency of a failed assessment. Obtaining less than 4 h of sleep in the last 24 h was associated with a relative frequency of a failed assessment of 2.99 times that of the assessments associated with 7–9 h of sleep. Obtaining more than 7–9 h of sleep was also associated with a higher failure rate (1.53 times relative frequency).

Multiple one-way analyses of variance (ANOVAs) were conducted to assess whether test results were impacted by crewmember position, time of day, timing of the assessment within the duty period, and self-reported sleep quantity in the last 24 h. A Bonferroni adjusted alpha value of 0.0125 (0.05/4) was used to account for the increased likelihood of Type I errors when performing multiple analyses with the four independent factors. The failure rate was 1.37 times higher for assessments that occurred between 12:00 am and 6:00 am (failure rate of 2.83%) as compared with other times during the day (failure rate of 2.07%) (*F*[1, 95 491] = 21.4, *p* < .001) (Table 1). The failure rate differed based on the timing of the assessment within the duty period (*F*[2, 95 490] = 44.4, *p* < .001), with the highest failure rate, 1.9 times higher than the start of shift (1.99%), associated with assessments completed during intermediate timepoints (failure rate of 3.72%) during the duty period (Table 2). For assessments taken at the start of shift, sleep quantity obtained in the last 24 h was found to be a significant factor, with each hour of sleep loss below 9 h of sleep accounting for an average 0.7% increase in the relative frequency of a failed assessment the (*F*[1, 54 612] = 168.1, *p* < .001). Figure 3 shows the relative frequency of failed assessments as a function of hours of sleep in the last 24 h. Obtaining less than 4 h of sleep (failure rate of 4.52%) in the last 24 h was associated with a frequency of a failed assessment 2.99 times higher compared with assessments reporting 7–9 h of sleep (failure rate of 1.51% for 7–9 h of sleep) (Table 3). Obtaining more than 9 h of sleep (failure rate 2.31%) was also associated with a higher failure rate 1.53 times higher compared to assessments reporting 7–9 h of sleep. Crewmember position was found to be a significant factor in explaining failure rate (*F*[5, 95 487] = 149.4, *p* < .001) with pilots having the lowest failure rate (0.83%), followed by mechanics (1.6%), flight paramedics (2.92%), flight nurses (3.5%), and OCC personnel (4.1%), and uncategorized (3.9%) (Table 4).

**Table 1.** Assessment results by assessment time

Time of day	Total tests	Tests ≥ 12 errors	% Tests ≥ 12 errors
06:00–23:59	86 936	1808	2.07%
00:00–05:59	8557	243	2.83%

**Table 2.** Assessment results by timing within duty period

Timing in shift	Total tests	Tests ≥ 12 errors	% Tests ≥ 12 errors
Start of shift	56 947	1133	1.99%
End of shift	31 608	660	2.09%
Intermediate time points	6938	258	3.72%

**Table 3.** Assessment results by reported prior sleep duration

Hours of sleep	Total tests	Tests ≥ 12 errors	% Tests ≥ 12 errors
<4 h	951	43	4.52%
4–7 h	26 323	622	2.41%
7–9 h	27 340	414	1.51%
>9 h	2333	54	2.31%

**Table 4.** Assessment results by crew role

Crew role	Total tests	Tests ≥ 12 errors	% Tests ≥ 12 errors
OCC personnel	6502	270	4.15%
Maintenance	7651	125	1.63%
Flight nurse	15 142	535	3.53%
Flight paramedic	19 596	558	2.85%
Pilot	40 883	339	0.83%
Uncategorized	5719	224	3.92%

## Discussion

The overall observed failure rate was 2.1%. Recognizing that failing a PVT may be associated with an increased risk of accidents or safety incidents, the organization’s objective during the next phase of implementation is to implement risk controls following each occurrence of a failed PVT result.

Examples of risk controls under consideration include taking the crewmember out of service and providing one or more hours of protected time off duty to obtain rest and restore alertness levels before resuming duty. Given the prevalence of assessments above the threshold, the operational impact of providing crewmembers this rest opportunity at a rate of approximately 2 out of 100 duty shifts is expected to have minimal impact on the operation and is therefore considered acceptable.

We believe that the PVT failure rate can be reduced over time by providing training to crewmembers about the critical importance of getting daily adequate rest and the use of personal fatigue countermeasures. Moreover, crewmembers who receive moderate scores (i.e. 8–11 total errors) will receive feedback following the assessment that promotes individual fatigue risk management best practices, such as allocating sufficient time for sleep during off-duty hours.

The PVT failure rate was 33% higher on assessments that occurred between 12:00 am and 6:00 am. Studies have shown that workers performing night duty predominately remain entrained to daytime schedules or experience only partial circadian adaptation to night work [13, 14]. Therefore, most crewmembers completing assessments at night are doing so at an adverse circadian phase. It is possible that the adverse circadian phase combines with other factors, such as sleep debt and the amount of time awake, to potentiate alertness deficits resulting in a higher failure rate [5]. The fact that the failure rate was 80% higher on assessments completed at intermediate time points during the duty period compared with assessments completed at the start or end of duty is also at least partially explained by circadian factors. A disproportionate number of assessments were completed between 12:00 am and 6:00 am (36.9%) compared with the start of duty (3.6%) and end of duty (12.4%).

Sleep quantity in the last 24 h demonstrated a linear relationship with PVT failure rate, with higher failure rates associated with less sleep. Obtaining fewer than 4 h of sleep resulted in a threefold increase in the failure rate. This result is consistent with the literature and provides evidence for the suitability of the failure threshold [4, 15, 16]. The failure rate also increases for sleep quantity in the last 24 h greater than 7–9 h. While this finding seems paradoxical, there are examples in the literature that long sleep durations may be associated with a higher prevalence of medical conditions [17, 18], which may impair alertness and contribute to higher failure rates.

Different failure rates across crewmember positions were expected as there are large differences in the scheduling approach for each position. For example, for a given week, the duty schedule for a pilot may involve 12-hour, daytime-only duty periods, whereas a flight nurse or flight paramedic’s schedule may involve 24-hour duty periods spanning both day and night. Additionally, flight nurses and flight paramedics frequently work second jobs on days off duty which may constrain their ability to get adequate recovery rest prior to their next scheduled duty. These position-related factors translate into differences in the timing and duration of sleep opportunities, circadian disruption, sleep debt, and duration of continuous wakefulness that affect failure rate on PVT assessments [4–6, 13–15]. Future studies should additionally examine interactions between independent factors such as, for example, the effect of sleep duration on PVT performance as a function of crewmember position.

This report provides evidence about the ecological validity and utility of the PVT as an objective quantitative measure of alertness to guide the application of risk controls in safety-critical operations. Having an estimate of a failure rate relative to a threshold of 12 errors on the PVT enables the implementation of active risk controls such as taking the crewmember out of service and providing one or more hours of protected time off duty to obtain rest and restore alertness levels before resuming duty. It is a demonstration of the real-world impact that extends from Dr Dinges’s legacy of scientific discovery.

## Data Availability

Due to the commercial restrictions of the research, all supporting data is not available.

## Notes

This paper is part of the David F. Dinges Festschrift Collection. This collection is sponsored by Pulsar Informatics and the Department of Psychiatry in the Perelman School of Medicine at the University of Pennsylvania.
